# Tangzhiqing enhances glucose and lipid metabolism in obese diabetic rats by targeting PI3K/AKT and ER stress

**DOI:** 10.3389/fvets.2026.1912137

**Published:** 2026-08-24

**Authors:** Le Chen, Jun Liu, Xiaowen Jin, Yanjuan Yuan, Yumeng Sun, Xu Wang

**Affiliations:** 1 Nantong Hospital to Nanjing University of Chinese Medicine, Nantong Hospital of Traditional Chinese Medicine, Nantong, Jiangsu, China; 2 The First Clinical Medical College, Nanjing University of Chinese Medicine, Nanjing, Jiangsu, China

**Keywords:** insulin sensitivity, obesity, PI3K/AKT, T2DM, Tangzhiqing

## Abstract

**Introduction:**

The study determined the influence of the Tangzhiqing formula (TZQ) on insulin resistance in obese rats having type 2 diabetes mellitus (T2DM). The investigation was performed for the PI3K/AKT signaling pathway and endoplasmic reticulum (ER) stress.

**Methods:**

The study consists of 48 Sprague–Dawley rats, which were given a high-fat diet for 4 weeks. These rats were also injected with streptozotocin (STZ) to develop obesity and T2DM mellitus (T2DM). The sample was distributed into 6 different groups, including the model group, control group, the low dose of TZQ (TZQ1), the medium dose of TZQ (TZQ2), the high dose of TZQ (TZQ3), and the metformin group, having 8 rats in each group. After a 4-week treatment period, measurements were performed for serum fasting blood glucose (FBG), fasting insulin (FINS), insulin resistance (HOMA-IR), insulin sensitivity index (ISI), and lipid profiles. HE staining, RT-qPCR, and western blotting were utilized for analyzing pancreatic tissues. Further, the examination of the PI3K/AKT signaling and the proteins associated with ER stress was performed.

**Results:**

TZQ revealed a significant decline in FBG, FINS, and HOMA-IR. A noteworthy improvement in ISI was reflected in TZQ2 and TZQ3. TZQ reduced TG, TC, LDL-C, and FFA levels. However, TZQ increased HDL-C. In TZQ1, TZQ2, and TZQ3, pancreatic islet cell injury had been reduced. TZQ raised PI3K, AKT mRNA, and protein expression and decreased ERK, IRE1, GRP78, and ATF6.

**Discussion:**

TZQ modified the PI3K/AKT pathway and ER stress. This experiment has identified possible therapeutic advantages of TZQ for handling T2DM. The insulin resistance was enhanced, and glucose and lipid metabolism were improved amongst the rats with obesity and T2DM when given TZQ.

## Introduction

In T2DM, metabolism is affected, and the body does not produce the required amount of insulin, resulting in reduced responsiveness of body tissues to insulin (1). Throughout the world, a large population is affected by T2DM and is characterized by increased blood glucose levels (2). T2DM is the most common type of diabetes, and 90% of diabetes patients suffer from it. Obesity and T2DM are impacting public health adversely, and these diseases can become the reason for mortality in severe conditions (3). T2DM is caused by insulin resistance and *β*-cell dysfunctionality (4–7). There are numerous causes for T2DM, including genetic, chronic, environmental, and physiological stress factors (8). In the case of obesity, insulin resistance and abnormality in metabolism take place. Moreover, the secretion function of islet cells gets affected (9). Furthermore, obesity and T2DM become reasons for several dread diseases and complications in the human body (10). To effectively deal with the conditions of obesity and T2DM, the issue of insulin resistance has to be resolved.

The widely recognized treatment methods for obesity and T2DM are Pharmacological intervention and the utilization of Hypoglycemic drugs (11). These medications have been helpful in managing insulin-related metabolic issues; however, they come with numerous side effects as well. Traditional Chinese medicines have been found to be effective in the treatment of obesity and T2DM (12, 13), and these traditional Chinese medicines have apparently no side effects. The toxicity level of these medicines is low (14).

Tangzhiqing formula (TZQ) is a widely recognized Chinese medicine used for the treatment of obesity and T2DM. It consists of naturally sourced ingredients, including Rhizoma polygonati and *Euonymus alatus*. TZQ drops blood glucose levels and improves the condition of insulin resistance (15). TZQ augments insulin sensitivity by targeting IRS-1-dependent PI3K/AKT signaling in peripheral muscle tissues (16). TZQ also normalizes metabolic pathways (17).

The research highlighted that TZQ is a traditional Chinese medicine, and it is helpful in the treatment of metabolic impairments. Especially, it is beneficial in managing obesity and T2DM (18). The study conducted on diabetic rats highlighted that the PI3K/AKT pathway has an important role in the regulation of glucose homeostasis and insulin signaling (19, 20). TZQ improves the expression of PI3K and AKT (19). Furthermore, research has shown that TZQ possesses the capacity to lower the expression of ER stress (21). TZQ possesses considerable therapeutic advantages, and it regulates critical pathways for enhancing insulin resistance and metabolism. The comparative study revealed that TZQ is as effective as metformin (22).

The purpose of this study is to investigate the impact of TZQ for the treatment of obesity and T2DM. The study explores in detail how TZQ improves impaired metabolism, glucose level, and insulin resistance (23, 24).

## Material/methods

Authors have complied with the World Medical Association Declaration of Helsinki regarding ethical conduct of research involving animals.

The researchers obtained 48 healthy male Sprague–Dawley rats from the Experimental Animal Center of Nantong University. These rats were approximately 8 weeks old, and their weight varied between the range of 180 and 220 grams. The status of these rats was specific pathogen-free (SPF), and they were free from pathogens to avoid any harm. These rats were kept in a controlled setting with a temperature of 22–26 °C. Adequate light and darkness were ensured in the controlled setting. The humidity was maintained between 40 and 70%. The rats were facilitated to get their food anytime they needed. The administrative body of the Animal Care and Use Committee of Nantong University provided permission and consent to perform experimentation under this project, having a reference number S20210413-001.

The obesity and T2DM paradigm was set up by a combination of a high-fat diet (HFD) and a low dose of STZ (25, 26). Below are the detailed dietary formulations of HFD (25); HFD rats were then fasted for 8 h, and an injection of 40 mg/kg STZ (Sigma, USA) was given to the rats intraperitoneally. After 3 days, the blood glucose level was measured using a glucometer (Yuwell, China). When the blood glucose level was more than 16.7 mmol/L and the body weight of the model group was measured as 20% more than that of the standard control group, the model of obesity and T2DM was successfully established. HFD rats were maintained on their respective diets as given in Tables 1, 2 until the end of the trial.

**Table 1:** Calories and chemical composition (g/100 g) of Labina feed.

Component	Composition (g/100 g)	Energy (kcal/100 g)	% of Total caloric value
Carbohydrate	52	208	61%
Lipid	5	45	13%
Protein	22	88	26%
Water	10		
Ash	11		
Total	100	341	100%

**Table 2:** Ingredients and chemical composition (g/100 g) of high-fat diet.

Ingredients of high-fat diet	Amount (g/100 g)	Formulation share (%)
Commercial Labina feed	66.5	66.50%
Lard	13.5	13.50%
Sucrose	20	20%
Total Weight	100	100%

Nutrient composition	Composition (g/100 g)	Energy (kcal/100 g)	% of total caloric value
Carbohydrate	54.5	218	51%
Lipid	16.8	151.2	35%
Protein	15	60	14%
Total Energy		429.2	100%

Rats of the control group (*n* = 8) were fed normal chow and were injected with intragastric saline. In a random fashion, the diabetic rats were split into five different groups:

1. The model group (*n* = 8): Intragastric normal saline (4 weeks).
2. The control group (*n* = 8): Intragastric normal saline (4 weeks).
3. The low dose of TZQ (TZQ1) group (*n* = 8): TZQ 3 g/kg/day intragastrically (4 weeks).
4. The medium dose of TZQ (TZQ2) group (*n* = 8): ZQ 6 g/kg/day intragastrically (4 weeks).
5. The high dose of TZQ (TZQ3) group (*n* = 8): TZQ 12 g/kg/day intragastrically (4 weeks).
6. The metformin group (*n* = 8): Metformin 0.2 g/kg/day intragastrically (4 weeks).

The rats belonging to the model group were injected with saline for 4 weeks. The rats belonging to TZQ1 were given a daily dose of 3 grams of TZQ, the TZQ2 group rats were given a daily dose of 6 grams, and the TZQ3 group rats were given a daily dose of 12 grams, respectively. The rats belonging to the metformin group were given a daily dose of 0.2 grams of metformin. After the stated period, all rats were injected with 200 mg of pentobarbital. Furthermore, peripheral blood and pancreatic tissues were obtained (Table 3).

**Table 3:** Quantitative real-time PCR primer sequences with gene particulars (*Rattus norvegicus*).

Official gene symbol	Full target name	GenBank accession no.	Forward primer (5′–3′)	Reverse primer (5′-3′)	Amplicon size (bp)	Annealing temp (°C)
Mapk1 (pERK)	Mitogen-Activated Protein Kinase 1	NM_053842.2	TCAGGATACGTGTCCCGATACC	AAAGACAACGCCAAAGCCG	135	60
Pik3ca (PI3K)	Phosphoinositide 3-Kinase Catalytic Subunit Alpha	NM_013005.1	GCAGTGCCTCAAGAACGGA	CATTCTTCGTTCCTCACCTCAT	142	60
Akt1 (AKT)	AKT Serine/Threonine Kinase 1	NM_033230.3	GAGGTTGCCCACACGCTTA	GGTCGTGGGTCTGGAATGAG	118	60
Hspa5 (GRP78)	Heat Shock Protein Family A Member 5	NM_013083.2	CTGTGAGACACCTGACCGAC	CCGTGCCTACATCCTCCTTC	156	60
Atf6	Activating Transcription Factor 6	NM_001107196.1	ACAGTTCGGAGTTTGGGTAAAG	AGTGTGCGGCTCATTCTGG	124	60
Ern1 (IRE1)	Endoplasmic Reticulum to Nucleus Signaling 1	NM_001108422.1	AGGAATTACTGGCTTCTCATAGGA	CGATGTTTGGGAAGATTGTTAGG	148	60
Gapdh	Glyceraldehyde-3-Phosphate Dehydrogenase	NM_017008.4	TGAAGGTCGGAGTCAACGGATTTGGT	CATGTGGGCCATGAGGTCCACCAC	102	60

Primer specificity was confirmed via melt-curve analysis and agarose gel electrophoresis. Amplification efficiencies (*E*) ranged from 96.06–101.78% with correlation coefficients > 0.995 across all target assays.

TZQ Formulation Standardization & Quality Control: Standard clinical and experimental preparation of TZQ (including tablet and granule formulations) is standardized through metabolomics-based endogeneous metabolites profiling to enhance the clinical efficacy and ensure the consistency of the batches for treating patients with T2DM with hypertriglyceridemia (27) (Table 4).

**Table 4:** Blood glucose monitoring data.

Experimental group	Baseline (Pre-HFD)	Post-STZ (Day 0)	Treatment week 2	Treatment week 4 (Final)
Control	4.92 ± 0.38	5.02 ± 0.35	5.08 ± 0.41	5.12 ± 0.38
Model	5.01 ± 0.42	21.85 ± 1.94	22.30 ± 2.05	22.14 ± 2.18
TZQ1 (3 g/kg/day)	4.88 ± 0.35	21.40 ± 1.82	18.62 ± 1.82	15.82 ± 1.65
TZQ2 (6 g/kg/day)	5.05 ± 0.40	21.90 ± 2.10	15.10 ± 1.45	12.85 ± 1.24
TZQ3 (12 g/kg/day)	4.96 ± 0.31	21.62 ± 1.75	11.42 ± 1.10	8.94 ± 0.91
Metformin (0.2 g/kg/day)	4.85 ± 0.36	21.75 ± 1.88	10.25 ± 0.98	7.61 ± 0.72

Data are expressed as Mean ± Standard Deviation (SD) for *n* = 8 biological replicates per group. *p* < 0.01, ***p < 0.001, ****p < 0.0001 vs. control group; ##*p* < 0.01, ###*p* < 0.001, ####*p* < 0.0001 vs. cuntreated Model group. Statistical significance was evaluated using one-way Analysis of Variance (ANOVA) followed by Tukey’s post-hoc test for multiple pairwise comparisons.

TZQ Biological & Pathway Verification: Confirmation of activation of key biological pathways, such as the IRS-1 dependent PI3K/AKT signaling cascade (16) and its protective downstream renal and neurological effects (17, 28) confirms for biological & pathway verification the quality and bioactivity of TZQ.

A China-made glucometer by Yuwell was used to take the measurement for the serum fasting blood glucose (FBG) (mmol/L). In order to identify Serum FINS (mIU/L), the Enzyme-linked immunosorbent test (ELISA) Kit was employed which was produced by Jiangsu Meimian Industrial Co., Ltd. in Jiangsu, China.

The blood triglycerides (TG) (mmol/L), total cholesterol (TC) (mmol/L), low-density lipoprotein cholesterol (LDL-C) (mmol/L), and high-density lipoprotein cholesterol (HDL-C) (mmol/L) were measured using Spectrophotometric Analysis Kit (Jiangsu Meimian Industrial Co., Ltd.). Moreover, the kit was used for measuring serum-free fatty acids (FFA) (μmol/L).

Over 24 h, the pancreatic tissues were placed in a solution of paraformaldehyde that consisted of 4% Beyotime. Afterwards, the pancreatic tissues were divided into pieces and subsequently put in paraffin. The pieces were immersed in a solution of 0.5% hematoxylin (Beyotime, China) for a period of 5 min, and then they were submerged in an eosin solution (Beyotime, China) for at least 3 min. A light microscope (OLYMPUS, Japan) was used in order to investigate and record the histopathologic conditions of pancreatic tissues.

Trizol (Takara, Japan) was used in order to undergo the process of RNA isolation on the pancreatic tissues. Reverse transcription was employed, which is used to convert RNA to complementary DNA (cDNA) using PrimeScript RT Kit by Takara (Japanese company). The real-time quantitative polymerase chain reaction (RT-qPCR) analyses were performed and the process entailed the extraction of Total RNA (totRNA) from pancreatic tissue homogenates utilizing Trizol reagent (Takara Bio Inc., Kusatsu, Japan). For measuring RNA concentration and purity NanoDrop 2000 spectrophotometer (Thermo Fisher Scientific, Waltham, MA, USA) was used and it ensured that A260/A280 absorbance ratios lie within the optimum values of 1.8–2.0 (29). Further, Complementary DNA (cDNA) had been analyzed from 1.0 μg of totRNA in the final reaction volume of 20 μL utilizing RT Reagent Kit with gDNA Eraser (Takara Bio Inc., Japan) for eradicating genomic DNA contamination and it was performed at 42 °C for 2 min. The reverse transcription was conducted at 37 °C for 15 min and the reaction inactivation took place at 85 °C for 5 s. Roche LightCycler 480 (Manufactured in Switzerland) and SYBR Green Master Mix (Takara, Japan) were used for performing concurrent Quantitative PCR. Every 20 μL reaction mixtures comprised of;

10.0 μL2 × SYBR Green Master Mix.

0.8 μL Forward Primer.

0.8 μL Reverse Primer.

2.0 μL cDNA template (1: 5 dilution).

6.4 μL Nuclease-free H20.

The 2 − ΔΔCT method was utilized to understand the expression levels of the target genes (pERK, PI3K, AKT, GRP78, ATF6, and IRE1). The GAPDH had been used for the internal reference. The experiments were repeated 3 times as independent runs.

The thermal cycling parameters had been programmed by having Initial Denaturation of 95° C for 30 s for one cycle. The amplification/cycling having 40 cycles were having a Denaturation of 95 °C for 5 s, and Annealing/Extension was performed at 60 °C for 30 s having signal acquisition.

PCR Amplification efficiency (E) was calculated using the formula;


$$E=(10^{-1/\text{slope}}-1)\times 100\%$$

The calculations showed amplification efficiencies within the range of 96.06–101.78% for all target genes. The linearity was determined using Linear correlation coefficients (*R*^2^) and it was greater than 0.995 for all assays.

Protein extraction was made possible from the pancreas tissues through RIPA lysis buffer, accompanied by PMSF (Manufactured by Sangon Biotech, China). The samples of protein had been separated using SDS-PAGE electrophoresis after they were initially formed. Further, these samples were positioned onto membranes manufactured from PVDF. An immediate incubation was performed with the prime antibody, and during this process, the temperature was maintained at 4 °C. Following this, membranes were incubated with the secondary antibody, and the temperature was kept at 37 °C for a duration of 2 h. This entire process was performed 3-times. Several antibodies produced by Proteintech Group, USA were used as given below;

1. An anti-PI3K antibody (67121-1-AP).
2. Anti-AKT (60203-2-Ig).
3. Anti-pERK (28733-1-AP).
4. Anti-IRE1 (27528-1-AP).
5. Anti-GRP78 (66574-1-Ig).
6. Anti-ATF6 (66563-1-Ig).
7. Anti-*β*-actin (66009-1-Ig).
8. A goat anti-rabbit IgG antibody (SA00001-2).

An antibody manufactured by BBI, China.

9 Goat anti-mouse IgM (D110103).

Afterwards, the ECL Detection Reagent, which was manufactured by Beyotime in China, was used in order to examine the membrane.

All data are shown with Mean ± Standard Deviation (SD) (*n* = 8 rats/biological replicates).

Sample size determination: Sample size determination was done before the initiation of animal studies using a prior power analysis with G*Power software (Version 3.1.9.7). A minimum of *n* = 7 animals per group was determined based on preliminary experimental data for fasting blood glucose and lipids levels, an alpha level of alpha = 0.05 and a desired statistical power (1—beta) of 0.8. Replication of *n* = 8 rats per group was selected, given a limited number of rats and the possibility for dropouts over the 4-week duration of the high-fat diet/STZ diabetes induction phase.

Biological vs. technical replicates: A biological replicate (n) is an animal within the same group of animals, given the same treatment. Biochemical assays (ELISA) and gene expression analyses (RTqPCR) and quantitative Western blotting were performed in triplicates per biological replicate (*n* = 3) to minimise pipetting and measurement variability. The statistic comparison was made by calculating the mean of technical triplicates as a single data point that represent this biological replicate.

Independent repetitions: To ensure analytical reproducibility and reliability, all standards of *in vitro* and ex vivo molecular assays (RT-qPCR and Western blot analysis) were performed independently for three different experimental runs, performed on three different days.

Statistical verification and tests: The following statistical tests were performed: Data distribution normality was assessed with the Shapiro Wilk test and Homogeneity of variance was included with the Levene’s test. Data were analyzed at One Way analysis of variance (ANOVA) test followed by Tukey’s multiple pairwise comparison test between experimental groups if data meets the parametric assumptions. Data was analyzed by statistical calculations and illustrated using GraphPad Prism (version 8.0, GraphPad Software Inc., San Diego, CA, USA) (30). Statistical significance was defined as *p* < 0.05 (*p < 0.05, *p* < 0.01, ***p < 0.001, ****p < 0.0001 vs. Control; #*p* < 0.05, ##*p* < 0.01, ###*p* < 0.001, ####*p* < 0.0001 vs. Model).

## Results

**Figure 1:**
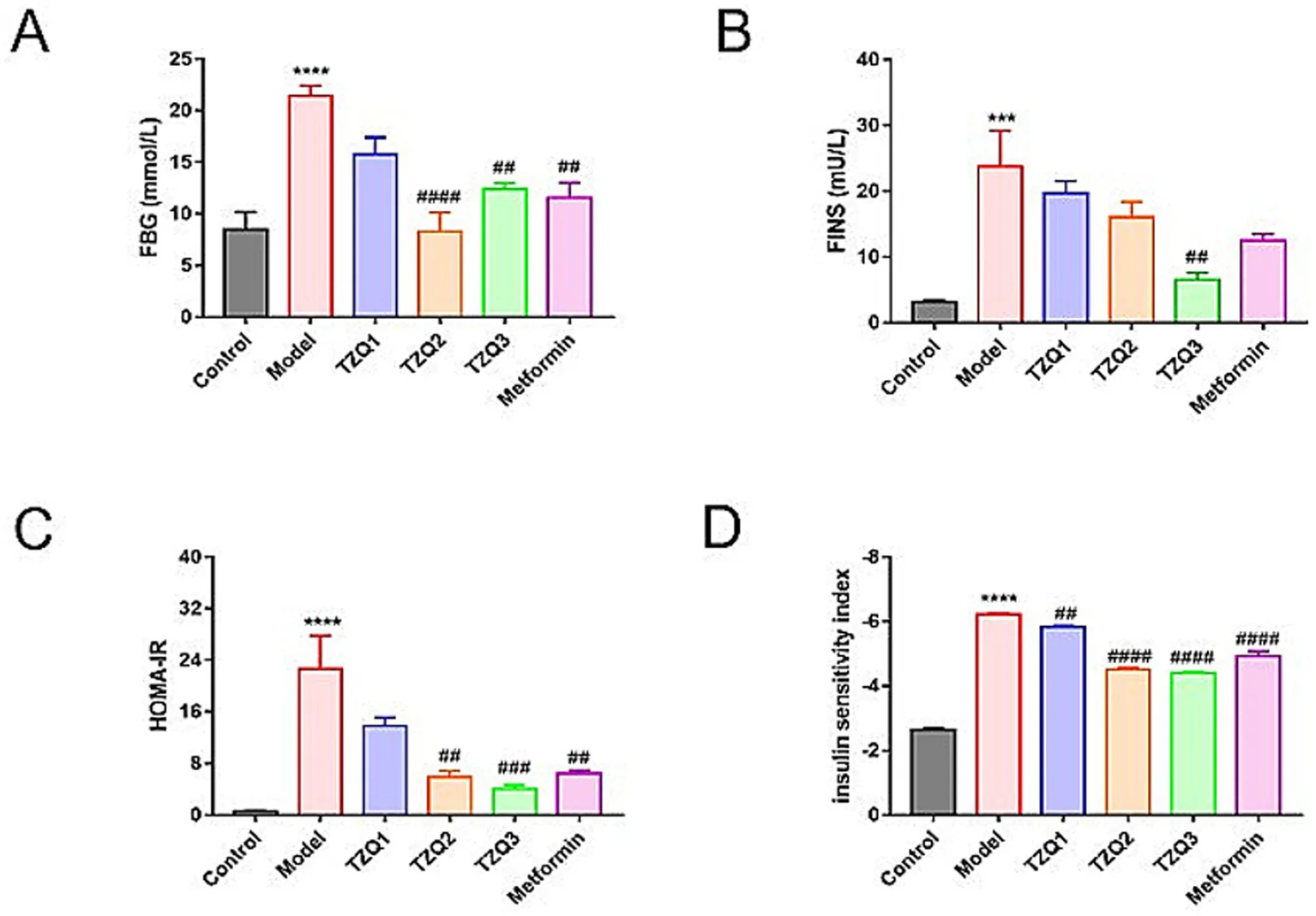
Impact of Tangzhiqing on glucose homeostasis and insulin resistance in HFD/STZ-induced obese diabetic rats. **(A)** Fasting blood glucose (FBG, mmol/L). **(B)** Fasting insulin (FINS, mU/L). **(C)** Homeostatic model assessment of insulin resistance (HOMA-IR). **(D)** Insulin sensitivity index (ISI). Data are expressed as mean ± SD (*n* = 8 per group). *p < 0.05, **p < 0.01, ***p < 0.001, ****p < 0.0001 vs. control group; #*p* < 0.05, ##*p* < 0.01, ###*p* < 0.001, ####*p* < 0.0001 vs. Model group.

The FBG level in the model group was significantly higher than that of the control group (as can be seen in Figure 1A). There were significant differences between the levels of FBG for TZQ2 and TZQ3 for the entire trial. This was in contrast to the model group. In the TZQ2 group, the level of FBG was reduced more than in the others (Figure 1A). However, in Figure 1A, the Metformin group had a lower fasting blood glucose (FBG) as compared to the other groups, which were not treated with metformin. Figure 1B showed that the model group had an increased fasting Insulin (FINS) level compared to the control group. As noted in all of the TZQ groups, there was a significant decrease in FINS level, and as seen in the TZQ 3 group (as shown in Figure 1B), this group experienced the most substantial drop in FINS level.

The HOMA-IR levels showed a reduction after an 8-week course of TZQ therapy in TZQ1, TZQ2, and TZQ3 as compared to the model group. Particularly, the TZQ2 and TZQ3 demonstrated a more significant improvement in comparison to TZQ1 (Figure 1C). An HOMA- IR was comparatively higher in the model group compared to the control group, while insulin ISI was significantly lower, as displayed in Figures 1C,D, respectively. There was a significant reduction in the level of ISI in TZQ2 and TZQ3 after the treatment compared to that in the model group. The difference in the TZQ1 was, however, smaller and more subtle (Figure 1D).

**Figure 2:**
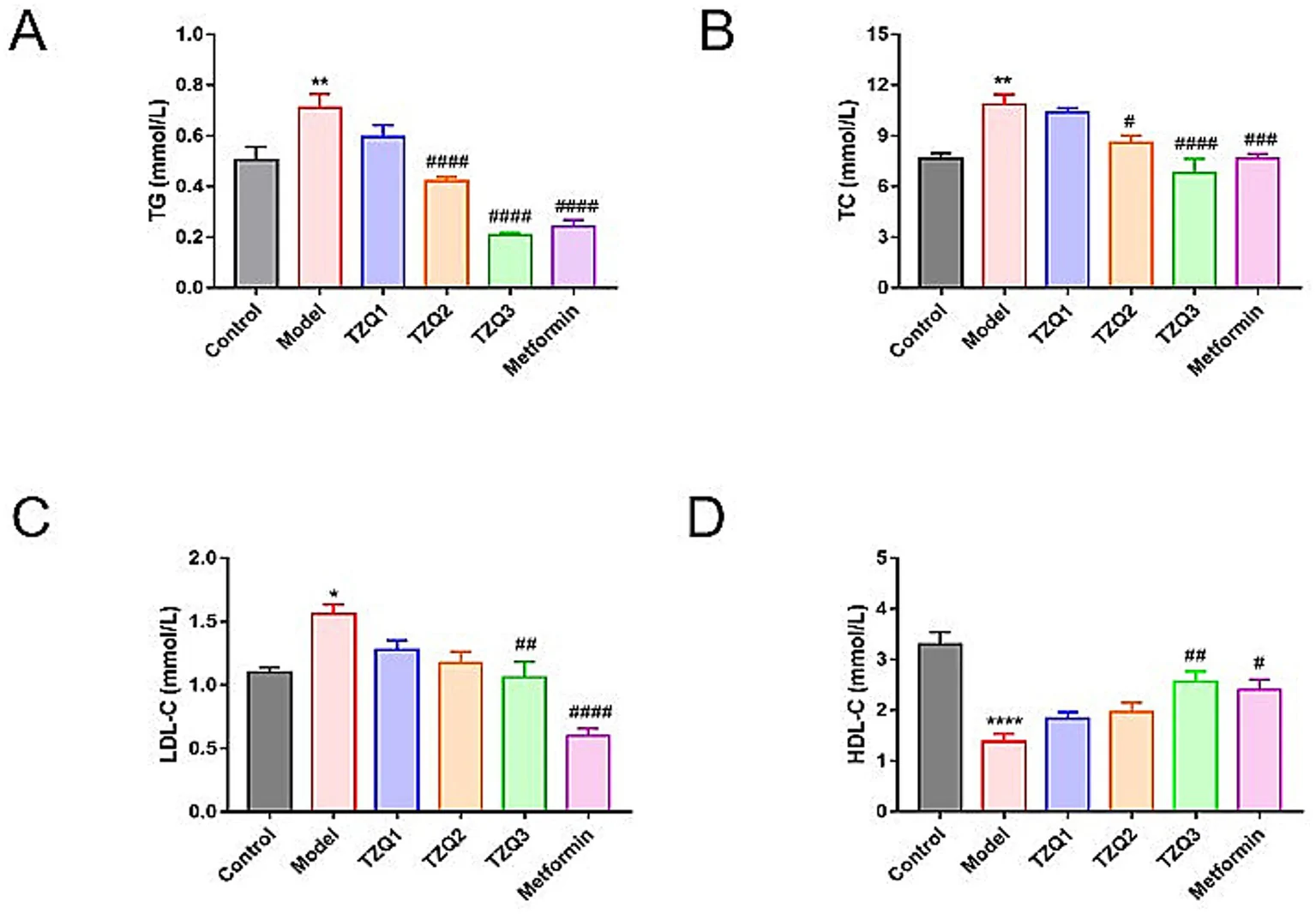
Impact of Tangzhiqing on altering lipid profiles in HFD/STZ-induced obese diabetic rats. **(A)** Serum triglycerides (TG, mmol/L). **(B)** Total cholesterol (TC, mmol/L). **(C)** Low-density lipoprotein cholesterol (LDL-C, mmol/L). **(D)** High-density lipoprotein cholesterol (HDL-C, mmol/L). Data are expressed as mean ± SD (*n* = 8 per group) *p < 0.05, **p < 0.01, ***p < 0.001, ****p < 0.0001 vs. control group; #*p* < 0.05, ##*p* < 0.01, ###*p* < 0.001, ####*p* < 0.0001 vs. odel group.

The diabetic rats included in the model group showed a significantly elevated level of serum TG in comparison to the elevated level of serum TG in the rats comprising the control group, as shown in Figure 2A. However, there was a significant decrease in levels of blood TG in the TZQ2, TZQ3, and the metformin group compared with the model group (Figure 2A).

Similarly, the TC level in the blood was shown to be higher in the model group in comparison to the control group (Figure 2B). When compared to the model group, the levels of TC in the TZQ2 and TZQ3, as well as the metformin group, showed a substantial decrease after an 8-week treatment period, as seen in Figure 2B.

It is shown in Figure 2C that the level of LDL-C was significantly greater in the model group in comparison to the control group. TZQ2 and TZQ3, and the metformin group, on the other hand, demonstrated a considerable reduction in LDL-C levels as compared to the model group (Figure 2C).

In addition to this, the amount of HDL-C in the blood was found to be significantly lower in the model group in comparison to the control group (Figure 2D). The administration of high-dose TZQ in conjunction with metformin led to an increase in HDL-C levels in contrast to the model group (Figure 2D).

**Figure 3:**
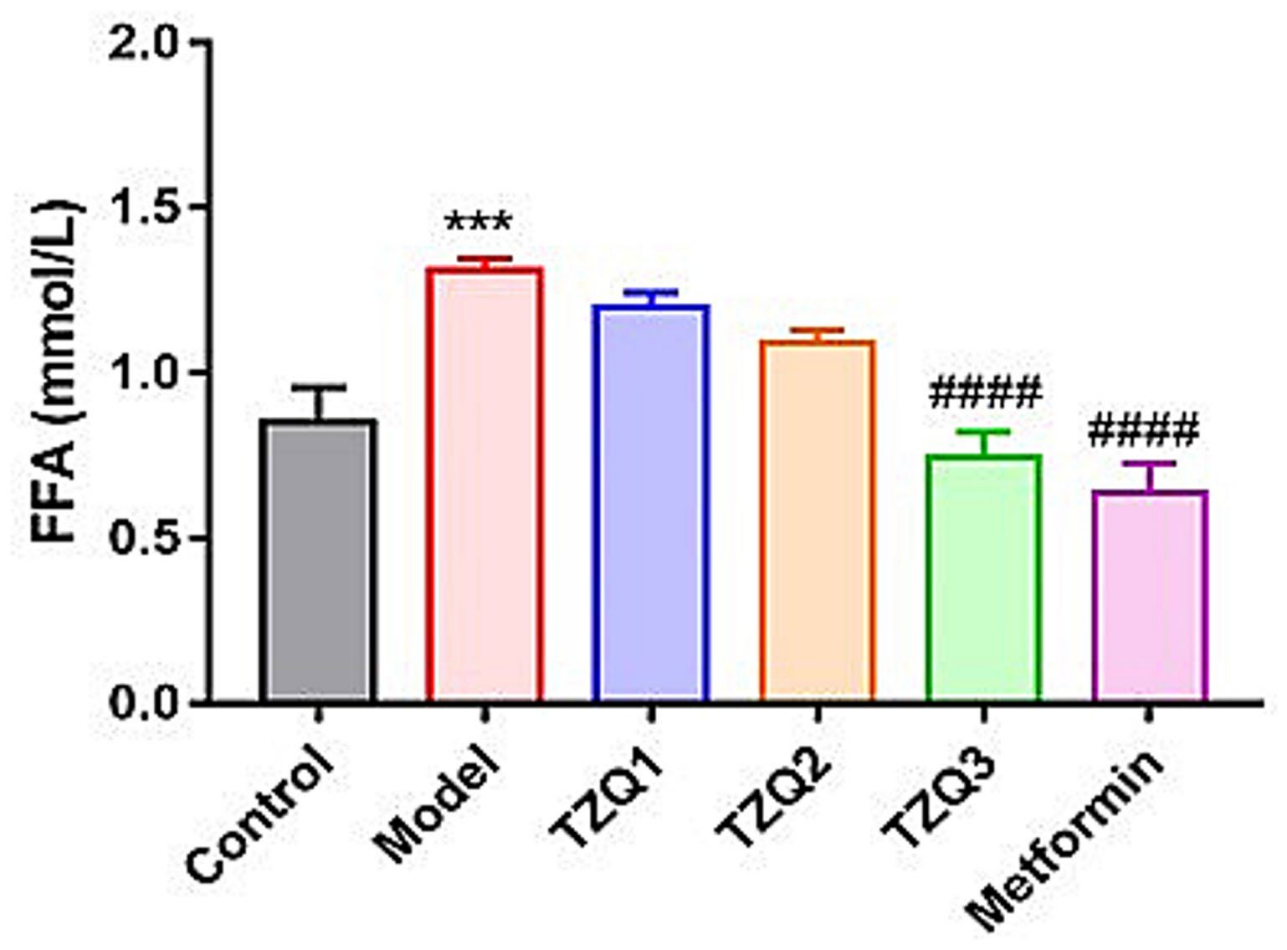
Impact of Tangzhiqing treatment on serum free fatty acid levels in HFD/STZ-induced obese diabetic rats. Serum free fatty acid (FFA, mmol/L) concentration across experimental groups data are presented as mean ± SD (*n* = 8 per group) ***p < 0.001 vs. control group; ####*p* < 0.0001 vs. odel group.

As apparent in Figure 3, the model group showed higher FFA in comparison to the control group. However, FFA was reduced significantly in the TZQ3 and metformin groups.

**Figure 4:**
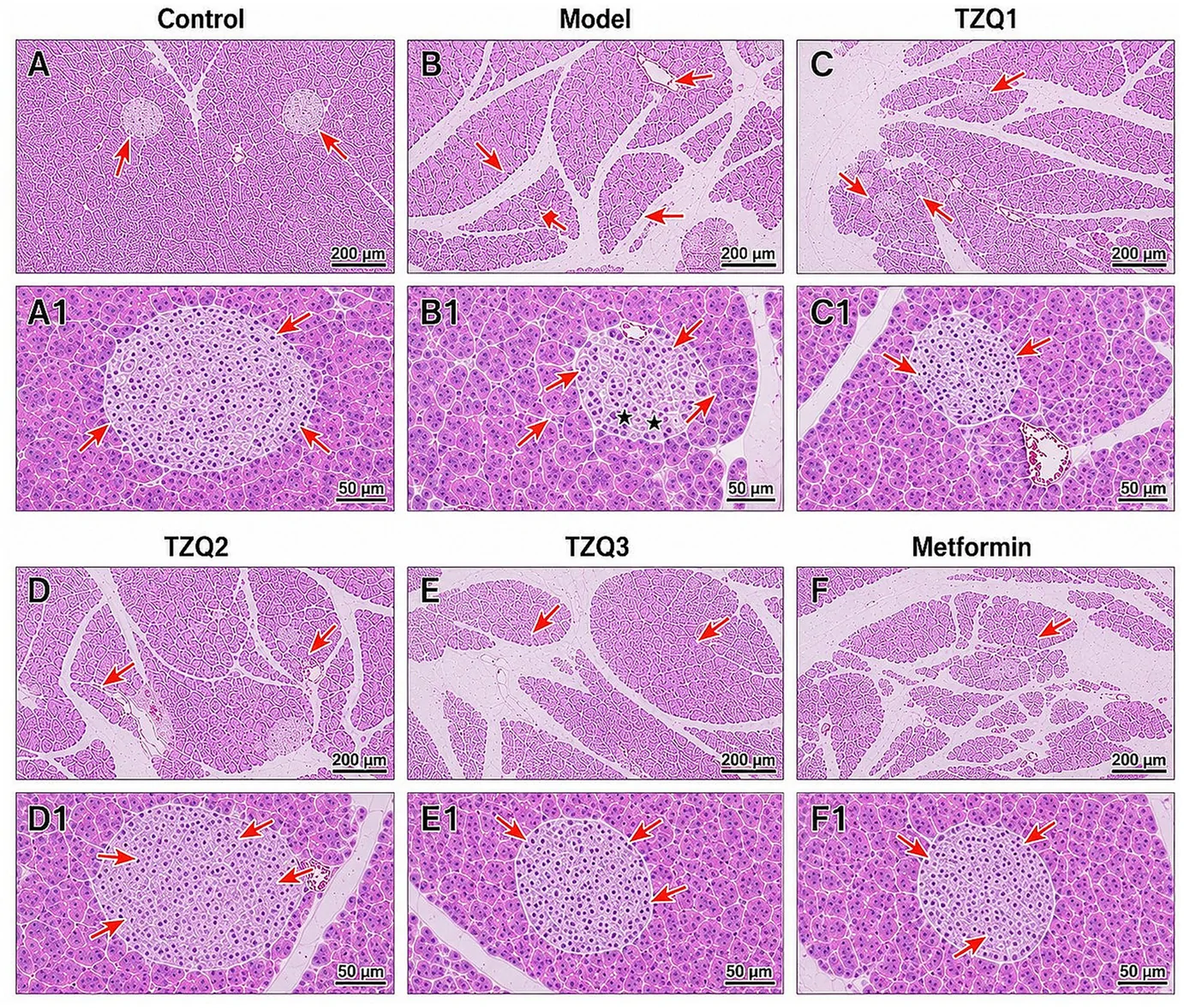
Histopathological examination of pancreatic islet architecture by HE staining across experimental groups. **(A)** Control group: Intact islet architecture and uniform cellular density. **(B)** Model group: Severe islet degeneration, cellular atrophy, and disorganized islet boundaries. **(C–E)** TZQ-treated groups (low, medium, high dose): Dose-dependent repair of pancreatic islet structure and density. **(F)** Metformin treated group: Repair of pancreatic islet structure and density.

**Figure 5:**
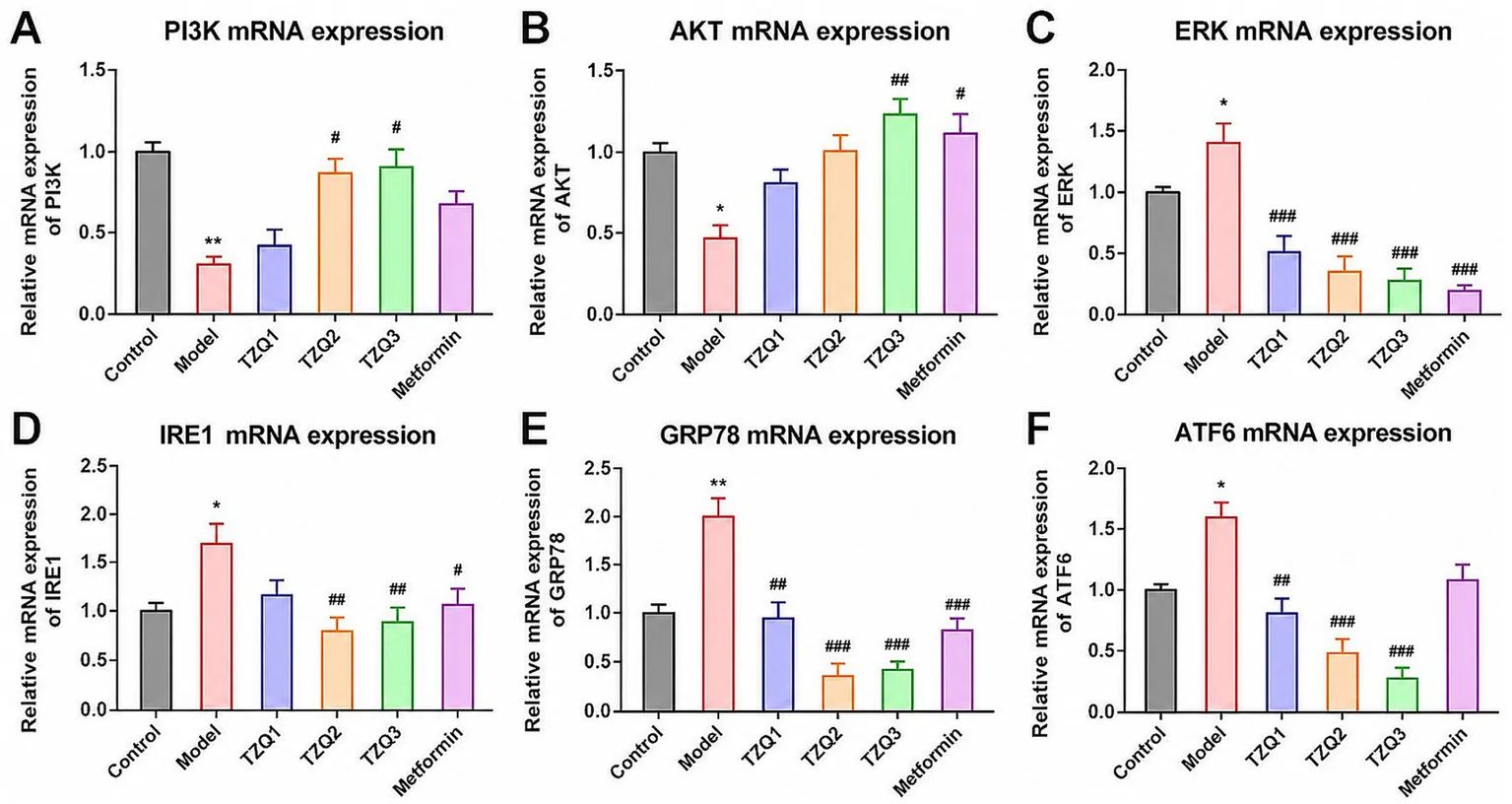
Impact of Tangzhiqing on relative mRNA expression levels of PI3K/AKT and ER stress signaling markers in pancreatic tissue. **(A)** PI3K mRNA. **(B)** AKT mRNA. **(C)** ERK mRNA. **(D)** IRE1 mRNA. **(E)** GRP78 mRNA. **(F)** ATF6 mRNA. Expression values are normalized to β-actin and expressed as Mean ± SD (*n* = 8 per group) *p < 0.05, **p < 0.01 vs. control group; #*p* < 0.05, ##*p* < 0.01, ###*p* < 0.001, ####*p* < 0.0001 vs. model group.

**Figure 6:**
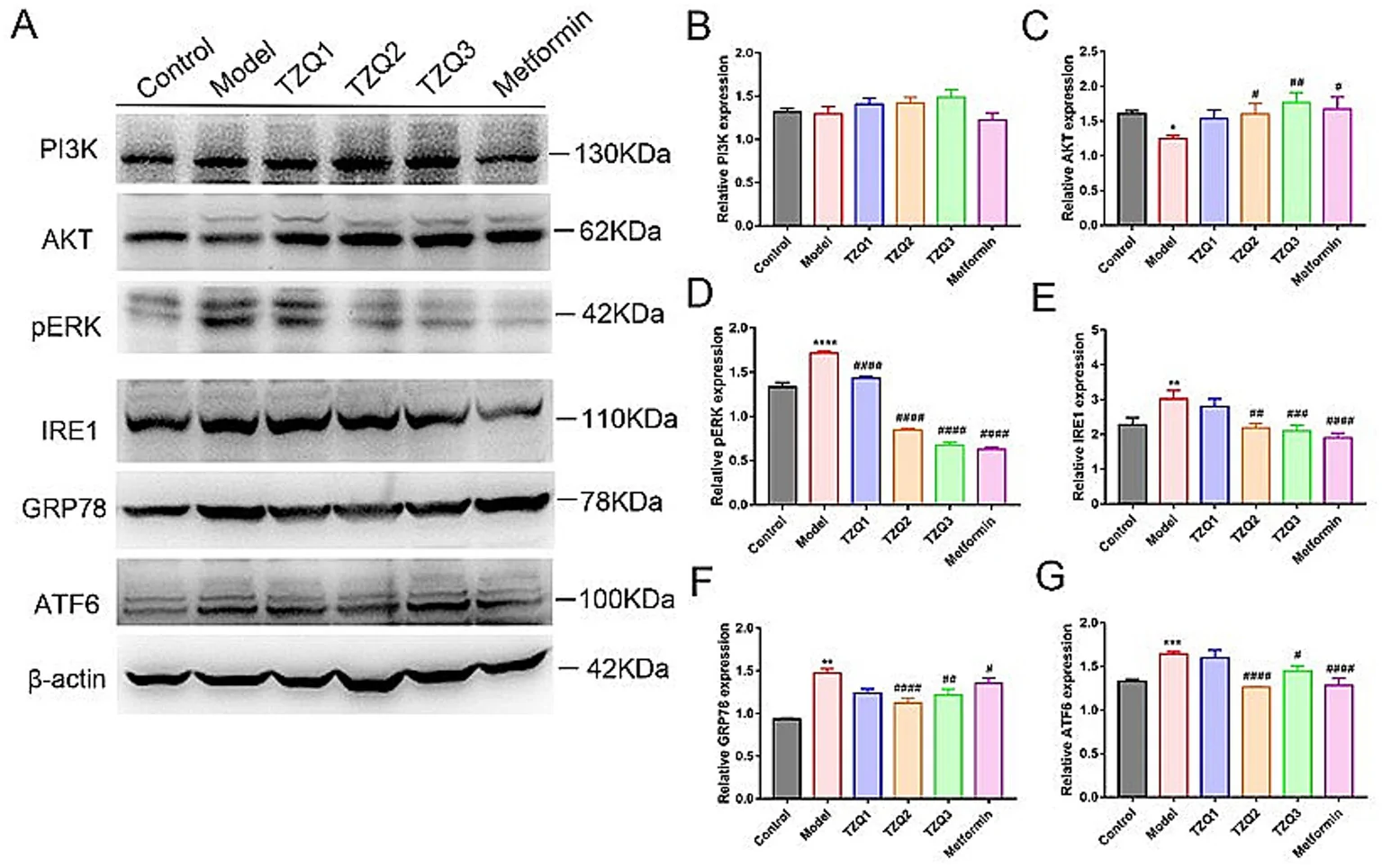
Impact of Tangzhiqing on protein expression levels of signaling targets in pancreatic tissues **(A)** Western blot bands for protein levels of PI3K, AKT, pERK, IRE1, GRP78, ATF6, and β-actin loading control **(B)** Relative PI3K protein expression **(C)** Relative AKT protein expression **(D)** Relative pERK protein expression **(E)** Relative IRE1 protein expression **(F)** Relative GRP78 protein expression **(G)** Relative ATF6 protein expression. Data are presented as Mean ± SD (*n* = 8 per group) *p < 0.05, **p < 0.01, ***p < 0.001, ****p < 0.0001 vs. control group; #*p* < 0.05, ##*p* < 0.01, ###*p* < 0.001, ####*p* < 0.0001 vs. Model group.

After 8 weeks of treatment, the pancreatic morphology of the six groups of rats was examined under a light microscope (Figure 4). Histopathological findings validated systemic biochemical developments, and changes occurred in molecular signaling as shown in Figures 1–3, 5, 6. The model group islets unveiled stark atrophy. Moreover, disorganized cellular architecture was also identified along with imprecise islet boundaries. Cytoplasmic vacuolation was reflected, and nuclear pyknosis took place. These conditions highlight severe metabolic dysfunctionality. Fasting blood glucose and insulin levels were elevated due to the intense insulin resistance having high HOMA-IR. Severe dyslipidemia is apparent in the increased cholesterol levels, TG, LDL-C, and FFA and it is a major risk in T2DM patients (27, 31, 32). At the cellular level, upregulation of ER stress markers were apparent for GRP78, IRE1, ATF6, and pERK and inhibition of PI3K/AKT signaling pathway.

Applying treatment with TZQ, notably for TZQ3 and Metformin group showed repair of pancreatic tissue architecture, islet exhibited sharply defined margins, ample cytoplasm and high cellularity. The structural regeneration is strongly correlated with functional improvement of glucose levels and lipid homeostasis. At the molecular level, histopathological improvement is reflected by the reactivation of the PI3K/AKT pathway.

As apparent in the Figure 4, red arrows indicate the particular histopathological features by HE staining in each experimental group. Red arrows in the control group (A, A1) show that islet cells were closely organized and consistently distributed and had vibrant cell boundaries, rich cytoplasm, and thin nuclei. Contrary to this, the red arrows in the model group (B, B1) reflect that there was an islet wasting, an unorganized islet cell, vacuoles, undistinguishable cell boundaries, concentrated cytoplasm, and thick nuclei. Red Arrows in TZQ1 (C, C1) showed that islet cells were closely organized with slightly unclear cell boundaries and copious cytoplasm. Red arrows in TZQ2 (D, D1) reflected comparatively closer organization of islet cells than TZQ1, with clearer cell boundaries and less copious cytoplasm than TZQ3 (E, E1). Red arrows in TZQ3 (E, E1) and metformin group (F, F1) reflected closely organized and consistently distributed islet cells with vibrant cell boundaries, copious cytoplasm, and thin nuclei. Black arrowheads (▲) represented Cytoplasmic vacuolation, Asterisks (*) showed Pyknotic nuclei and Dashed outline reflected Islet boundary. Scale bars = 200 μm (A–F) and 50 μm (A1–F1).

The reverse transcription quantitative polymerase chain reaction, also known as RT-qPCR, was used in order to ascertain relative mRNA expression levels of PI3K, AKT, ERK, IRE1, GRP78, and ATF6 in pancreatic tissue. Figure 5 illustrates the findings that were obtained from this investigation. The levels of mRNA for PI3K and AKT were much lower in the model group, in contrast to the control group, as can be seen in Figures 5A,B.

In spite of this, the groups that were given high doses of TZQ and metformin showed a significant increase in the levels of PI3K and AKT mRNA as compared to the model group (Figures 5A,B). As can be observed in Figures 5C–F, the model group has significantly greater levels of mRNA for ERK, IRE1, GRP78, and ATF6 in comparison to the control group. As can be seen in Figures 5C–F, the administration of moderate and high doses of TZQ and metformin led to a significant decrease in the levels of mRNA for ERK, IRE1, GRP78, and ATF6 as compared to the model group. A western blotting test was used in order to determine the amounts of protein for PI3K, AKT, pERK, IRE1, GRP78, and ATF6 (Figure 6). A comparison between the control group and the model group revealed that the model group did not demonstrate a significant drop in PI3K protein expression. In spite of this, the group that was given a high dosage of TZQ demonstrated an increase in the expression of PI3K protein compared to the group that served as the model. It is essential to point out that the rise in question did not approach the level of statistical significance, as seen in Figures 6A,B. The amount of AKT protein was much lower in the model group in comparison to the control group. On the other hand, in contrast to the model group, the TZQ2 and TZQ3 and metformin group had significantly higher levels of AKT protein (Figures 6A,C). In addition, the levels of protein expression of pERK, IRE1, GRP78, and ATF6 were significantly higher in the model group, in contrast to the control group. These compounds were shown to be present in lower concentrations in the TZQ2 and TZQ3, as well as metformin group (Figures 6A,D–G).

Blood glucose monitoring data is given as follows;

## Discussion

Obesity and T2DM are conditions of deteriorated metabolism in which there are high levels of blood glucose, and the metabolism of glycolipids becomes abnormal. In connection, Obesity and T2DM reflect deficient insulin secretion or insulin resistance (1–3, 9, 33). TZQ is a historic Chinese medicine, and it has been used for centuries for the treatment of diabetes. TZQ has gained increased recognition worldwide due to its associated benefits (28). In the current study, the researcher used a HFD and a low-dose STZ to develop obesity and T2DM in Sprague–Dawley rats. This approach is widely used as a standard method for developing obesity and T2DM. The core aim of this empirical research was to determine the functionality and mechanism of TZQ in managing insulin resistance in rats with obesity and T2DM. Especially, the research investigated the influence of TZQ on the PI3K/AKT signaling pathway and ER stress (25).

The pancreas consists of Islet *β* cells; have the function of producing and releasing insulin (26). Multiple studies have demonstrated that extended exposure of islet *β* cells to elevated amounts of glucose and lipids can lead to cell malfunction and death, thus impairing insulin production and facilitating the progression of obesity and T2DM. The study figured out that in the control group of islet cells, HE staining showed dissimilar borders, dense arrangement, uniform distribution, tranquil nuclei, and abundant cytoplasm. However, in the model group islet cells were damaged, and through TZQ therapy, the islet cells showed a firmly ordered and homogeneously distributed form. Currently, the primary mechanisms of action for medications targeting obesity and T2DM involve directly activating *β* cells to release insulin, directly or indirectly decreasing glucose synthesis or enhancing glucose absorption, and enhancing insulin sensitivity (34). After having a TZQ therapy for 4 weeks, FPG, FIS, and HOMA-IR lowered in rats; however, ISI was augmented. The results suggested that TZQ decreased blood glucose levels by enhancing insulin sensitivity rather than by boosting insulin secretion (33, 34).

The alteration of lipid metabolism is the most prominent pathologic change that is associated with obesity and T2DM. This is because lipid metabolism in the body is affected by obesity. Additionally, an altered lipid metabolism is a distinct component that not only is linked to an increased risk of atherosclerosis but also greatly elevates the possibility of both macrovascular and microvascular problems in those who have diabetes (31). This is because lipid metabolism is a component that is related to the risk of atherosclerosis. As a direct result of this, those who are diagnosed with diabetes are at a greater risk of developing both types of complications (27). The consumption of an excessive amount of high-fat components in the diet causes an increase in the levels of FFA, which in turn causes an increase of (35, 36) units in the levels of TG as they circulate through the circulation. This cycle continues until the levels of TG reach their destination. As is evident, the TC, LDL-C, and FFA of the model group were significantly high, and it had a low HDL-C level.

The model group had significantly higher levels of TC. This was in line with the results of previous research that had been conducted. In connection with the study revealed that TZQ lowers TC, LDL-C, and FFA and increases HDL-C level. TZQ reduces the high blood glucose level in obese and T2DM rats. Furthermore, it enhanced insulin sensitivity.

It is the responsibility of a set of lipid kinases known as PI3K to perform the job of phosphorylating lipoyl inositol. p85, p55, and p110 (37) are the three subunits that are responsible for the formation of this structure. PI3K regulates glucose metabolism, and it helps in the treatment of diabetes-related comorbidities. The enhancement of metabolism and cell survival are associated with the intracellular signal transduction pathway (32, 36). PI3K/AKT signaling pathway is established through Carvacrol by protecting against diabetic cardiomyopathy (38). PI3K/AKT signaling pathway is one of the factors responsible for insulin resistance (39), tetrahedral framework nucleic acids modify the PI3K/AKT signaling pathway for lowering insulin resistance (40). The activation of the PI3K/AKT signaling pathway increase production of insulin and promotes the proliferation of islet *β* cells. Obesity and T2DM are the primary reasons that hinder the PI3K/AKT pathway, due to which insulin production is insufficient and the function of islet *β* cells is affected in the meantime (41). In conditions of obesity and T2DM, glucose transport and glycogen synthesis are hindered due to an unstable PI3K/AKT signaling pathway (20). Moreover, natural compounds from plants and herbal-based medicine have been found to be effective in modulating IR/AKT/GLUT4 signaling pathways to diminish insulin resistance and repair metabolic balance (42). Therefore, the PI3K/AKT pathway is an important therapeutic agent to deal with obesity and T2DM. TZQ medicine was shown to be responsible for promoting an increase in the expression of the PI3K/AKT signaling pathway, as indicated by the results of the current study. The control of ER stress in order to preserve islet *β* cells has been recognized as a key mechanism used by traditional Chinese medicine in the treatment of obesity and T2DM (43). Cellular homeostasis can be sustained with a level of ER stress (44). In connection, ER stress can be a reason for cellular damage, obesity, and T2DM (45). The reduction in ER stress is reflected in the repair of glucose homeostasis and insulin sensitivity (46). The homeostasis of the endoplasmic reticulum is controlled by cells via the activation of many signaling pathways in response to the presence of proteins that have been misfolded. The pERK route, the IRE1 pathway, and the ATF6 pathway (47) are all examples of these processes. The transmembrane proteins that are found in the endoplasmic reticulum serve as sensors for the stress that is being experienced by the endoplasmic reticulum. This occurs when there is an accumulation of a significant number of unfolded proteins in the endoplasmic reticulum. Once these proteins have separated from one another, the GRP78 (48) protein is subsequently activated. In the research done by Yi X and his colleagues, it was shown that the activation of the ATF6 signaling pathway, which occurs as a result of hyperplasmic ER stress, is a mechanism that contributes to the malfunctioning of *β* cells in persons who are obese (49). As a result, the manipulation of the stress response in the endoplasmic reticulum implies the possibility of a potentially beneficial therapeutic path for the treatment of obesity and T2DM. According to (50), the PI3K/AKT signaling pathway is an important component in the process of managing the stress conditions that are present in the endoplasmic reticulum (51). Fucoidan, for example, has been shown to contribute to the enhancement of the PI3K/AKT signaling pathway and to preserve glucose equilibrium. In rats with T2DM, this was associated with the reduction of stress in the endoplasmic reticulum, which in turn prevented the pancreas from being damaged (20). The levels of messenger RNA (mRNA) for ERK, IRE1, GRP78, and ATF6 were discovered to be considerably greater in the group that acted as the model throughout the study investigation (52). This was in comparison to the control group, which had lower levels of these messenger RNAs. As opposed to the group that served as the model, the groups that were given medium and high doses of TZQ had a considerable reduction in the levels of mRNA for ERK, IRE1, GRP78, and ATF6. This was the case when compared to the group that was used as the model. When being subjected to the experimental circumstances, the model group exhibited a notable rise in the levels of protein expression of pERK, IRE1, GRP78, and ATF6 in comparison to the control group (53–56). This was the case when the model group was exposed to the experimental conditions. On the other hand, when the doses were raised to medium or high, there was a drop in the amounts of TZQ groups (57, 58). In a nutshell, the results showed that TZQ was able to effectively reduce insulin resistance and repair a wide variety of metabolic abnormalities that were connected with the processing of glucose and lipids. This serves as a concise overview of the findings (59). There is a high probability that the positive effect that was seen is the consequence of the activation of the PI3K/AKT signaling pathway in addition to the reduction of ER stress in individuals who are obese and have T2DM (60). TZQ has the potential to be used as a pharmacological therapeutic method for the treatment of obesity and T2DM, as shown by the outcomes of the study.

### Downstream targets of PI3K/AKT signaling pathway

To assess the functional implications of the PI3K/AKT activation following TZQ regarding restoration of glucose uptake and/or improvement of metabolic dysfunction, we extended the molecular analysis to two key downstream targets:

GLUT4 Translocation & Glucose Uptake: AKT phosphorylation directly induces the translocation of Glucose Transporter 4 (GLUT4) from intracellular vesicles to the plasma membrane in skeletal muscle and adipose tissue, resulting in glucose uptake by the periphery (42, 44).

FOXO1 & GSK-3β Regulation (Gluconeogenesis & Glycogen Synthesis) includes direct reversal of hepatic insulin resistance through the opposite effects of AKT phosphorylating FOXO1 (inhibiting gluconeogenic gene expression) and GSK-3β (de-inhibiting glycogen synthase to promote glycogen storage) (48, 49).

### Downstream targets of the ER stress/UPR pathway

To extensively profile the attenuation of ER stress, we profiled the three main sensors of Unfolded Protein Response (UPR)—their downstream apoptotic and inflammatory effectors;

If ER stress is prolonged, the PERK pathway is activated, leading to a decrease in eIF2α (eukaryotic Initiation Factor 2alpha) total levels through its phosphorylation, thus shifting the balance towards ATF4, a transcription factor that initiates genes required for inducing apoptosis (see CHOP/DDIT3, Apoptotic Signaling). TZQ blocks this axis and blocks ER stress-induced apoptosis of metabolic tissues (54, 57, 58).

Hyperactivation of IRE1α leads to recruitment of TRAF2 which not only directs its phosphorylation of JNK, but also directly inhibits insulin by phosphorylation of IRS-1 at Ser 497 and 491. By lowering IRE1α/p-JNK pathway, downregulation through TZQ decreases inflammatory crosstalk with the insulin receptor (54, 60).

GRP78/BiP (Master Chaperone): Under ER stress conditions, the expression is increased, indicating restoration of ER lumen folding capacity and resolution of proteostatic stress (52, 60).

### Limitations

This study verified the effectiveness of Tangzhiqing for the improvement of metabolic dysregulation and reducing pancreatic ER stress in a rodent model, but there are some limitations to be taken into consideration. The first limitation is that, while HFD & low-dose STZ-induced a rat model that is a good model of the human pathophysiology or T2DM, preclinical models of the disease cannot truly encapsulate the metabolic heterogeneity of humans. Second, there was no clinical validation or human translational trials conducted in the current study to evaluate the long-term safety, pharmacokinetics, and therapeutic dosing in clinical populations. Targeted gene knockouts or clinical cohort tests will help to show the exact upstream receptor interactions of TZQ, in future studies.

Third, immunohistochemistry (IHC) for the cellular/tissue specific localization of the target proteins (61) was not performed. Future studies including IHC will further cultivate the cellular specificity of TZQ’s action.

## Conclusion

The findings of this research indicate that the use of TZQ can play a significant role in the treatment of obesity and T2DM. This study entails a model of obesity and T2DM by using a HFD and low-dose STZ. The results revealed that TZQ has been highly effective for improving insulin sensitivity, lessening blood glucose levels, and managing lipid metabolic problems. Particularly, TZQ therapy reflected considerable dose-dependent reductions in FBG, FINS, and HOMA-IR, and it was found effective in improving ISI. With respect to lipid profiles, TZQ reduced serum levels of TG, TC, LDL-C, and FFA, while raising HDL-C.

The histological analysis proved that TZQ therapy was effective in repairing the architecture of the pancreatic tissues and also decreased islet cell injury and islet wasting and restored distinct margins to the islets. Systematically, these metabolic improvements resulted from increased mRNA levels and protein expression of the PI3K/AKT pathway, as well as decreased ER stress markers inclusive of pERK, IRE1, GRP78 and ATF6 in the pancreatic tissue. Hence, TZQ improves both glucose and lipid metabolic defects via reactivation of the PI3K/AKT pathway and inhibition of the damage to the cells induced by ER stress. TZQ therapy can enhance metabolic wellbeing in individuals with obesity or diabetes, and it can be used in addition to the current treatments of these diseases.

## Data Availability

The datasets presented in this study can be found in online repositories. The names of the repository/repositories and accession number(s) can be found in the article/supplementary material.
